# Association between Smoking during Pregnancy and Short Root Anomaly in Offspring

**DOI:** 10.3390/ijerph182111662

**Published:** 2021-11-06

**Authors:** Yuki Sagawa, Takuya Ogawa, Yusuke Matsuyama, Junka Nakagawa Kang, Miyu Yoshizawa Araki, Yuko Unnai Yasuda, Tsasan Tumurkhuu, Ganjargal Ganburged, Amarsaikhan Bazar, Toshihiro Tanaka, Takeo Fujiwara, Keiji Moriyama

**Affiliations:** 1 Department of Maxillofacial Orthognathics, Graduate School of Medical and Dental Sciences, Tokyo Medical and Dental University (TMDU), Tokyo 113-8549, Japan; sagamort@tmd.ac.jp (Y.S.); j-kan.mort@tmd.ac.jp (J.N.K.); miyujaga.pote@gmail.com (M.Y.A.); yuko.unnai.yasuda@gmail.com (Y.U.Y.); k-moriyama.mort@tmd.ac.jp (K.M.); 2 Department of Global Health Promotion, Graduate School of Medical and Dental Sciences, Tokyo Medical and Dental University (TMDU), Tokyo 113-8549, Japan; matsuyama-thk@umin.org (Y.M.); fujiwara.hlth@tmd.ac.jp (T.F.); 3 Department of Orthodontics, School of Dentistry, Mongolian National University of Medical Sciences, Ulaanbaatar 14210, Mongolia; tsasant@gmail.com (T.T.); ganjargal@mnums.edu.mn (G.G.); 4 Department of Prosthodontics, School of Dentistry, Mongolian National University of Medical Sciences, Ulaanbaatar 14210, Mongolia; amarsaikhan@mnums.edu.mn; 5 Department of Human Genetics and Disease Diversity, Graduate School of Medical and Dental Sciences, Tokyo Medical and Dental University (TMDU), Tokyo 113-8549, Japan; ttana.brc@tmd.ac.jp; 6 BioResource Research Center, Tokyo Medical and Dental University (TMDU), Tokyo 113-8549, Japan

**Keywords:** tobacco, tooth development, dental morphology, dental public health, epidemiology, community dentistry

## Abstract

Short root anomaly (SRA) is a dental anomaly with short dental roots and its pathogenesis is poorly understood. This study investigated the association between maternal smoking during pregnancy and SRA in offspring. A survey was conducted on 558 children aged 8–16 years from two public schools in Ulaanbaatar, Mongolia. SRA was diagnosed using cases with a root-crown ratio of maxillary central incisors of ≤1.0. A questionnaire survey was conducted to assess maternal lifestyle habits. Multiple logistic regression was used to analyse the association between maternal smoking during pregnancy and SRA in offspring after adjusting for possible confounders. The prevalence of SRA in these children was 14.2%. Children whose mothers smoked from pregnancy to date were found to be 4.95 times (95% confidence interval [CI]: 1.65–14.79) more likely to have SRA than those whose mothers never smoked, after adjusting for possible confounders. Additionally, children whose mothers had been exposed to passive smoking during pregnancy were found to be 1.86 times (95% CI: 1.02–3.40) more likely to have SRA than those whose mothers had not been exposed to passive smoke. Our population-based study suggests that maternal and passive smoking exposure during pregnancy can affect tooth root formation in children.

## 1. Introduction

A tooth is composed of two main anatomical elements, the crown and the root. In particular, the root not only supports the crown but also transmits occlusal forces through the periodontal ligament. Furthermore, it also plays an important role as a structure containing the neurovascular bundle that supplies blood, nutrition, and sensation to the crown. The process of root formation is significantly complicated and involves various factors that perform crucial functions in regulating epithelial-mesenchymal interactions [1]. Disruption of this process by genetic and/or environmental factors through prenatal and postnatal periods affects root formation.

Short root anomaly (SRA) was defined by Lind as a dental disorder affecting tooth root formation [2] when the root-crown (R/C) ratio was ≤1.0 [3], and 70% of the SRA was found in the maxillary central incisors [3]. The incidence rates of SRAs are 1.3–2.4% in Caucasians [3,4] and 10% in Japanese individuals [5], suggesting that there may be a difference in incidence by race. The tooth morphology of the population of Northeast Asian countries, including Mongolia, Korea, and Japan, is distinct, such as shovel-shaped teeth and smaller R/C ratios [6,7,8,9]. The etiologic factors of SRA remain largely unknown, but SRA occurs when the root formation is affected by genetic factors [10,11] or environmental factors such as trauma, chemotherapy, and radiation therapy [12,13,14].

Exposure to smoking or passive smoking is one of the environmental factors associated with a variety of health problems [15]. In recent years, the use of heated tobacco products (HTP) has become widespread because they are assumed to be less harmful to the body than paper cigarette. However, in addition to its effects on oral cells [16], HTP use has also been associated with the risk of gestational hypertension (HDP) and low birth weight (LBW) [17]. Hence, the impact of smoking has received renewed attention and is becoming a new public health concern.

Maternal smoking during pregnancy has its effect not only on cleft lip and/or palate but also on missing teeth in the offspring [18,19,20]. It may also affect various organs of the maxillofacial region formed by epithelial-mesenchymal interactions during the embryonic period [21]. Furthermore, maternal smoking during pregnancy also has a wide range of systemic effects such as respiratory disease, heart disease, chronic kidney disease, and allergies in children, and the prenatal effects may continue for a long time after birth [22]. Therefore, we hypothesize that maternal smoking has some effects on root formation, which is thought to be formed after birth.

SRA is a risk factor for root resorption in orthodontic treatment [23]. Furthermore, such teeth have a poor prognosis when used as abutment in prosthetic treatments due to lack of stability [24]. Therefore, elucidating the risk factors of SRA and establishing a preventive method can be significant in dental treatment. To the best our knowledge, no previous population-based study has examined the association between SRA and prenatal environmental factors such as maternal smoking. Therefore, our cross-sectional study with population-based samples aimed to test the hypothesis that maternal smoking during pregnancy would lead to SRA in offspring.

## 2. Materials and Methods

### 2.1. Design and Settings

This study used cross-sectional data derived from the longitudinal population-based survey ‘Craniofacial Collaborative Research’. The study was conducted by a team at Tokyo Medical and Dental University (TMDU) and the Mongolian National University of Medical Sciences (MNUMS). This article was structured according to the ‘Strengthening the Reporting of Observational Studies in Epidemiology’ guidelines for cross-sectional studies. The study field was Ulaanbaatar, the capital city of Mongolia. Ulaanbaatar was chosen as a convenient location for the study in an effort to increase the number of study participants; it is estimated that nearly half of the country’s total population lives there, and more than one-third of the school children study there. The survey was conducted in two public schools, the largest in each of the two largest districts in Ulaanbaatar (i.e., Bayanzürkh and Songino Khairkhan). The study involved children (aged between 5 and 16 years), randomly selected from each year group from the 1st to 10th grades. Further details concerning the survey have been published elsewhere [25,26,27,28]. This study was approved by the Ethical Review Board of the MNUMS (No. 13-12/1A) and TMDU (No. D2013-071). Informed consent for this study was obtained from caregivers of children.

### 2.2. Sampling and Recruitment

**Figure 1:**
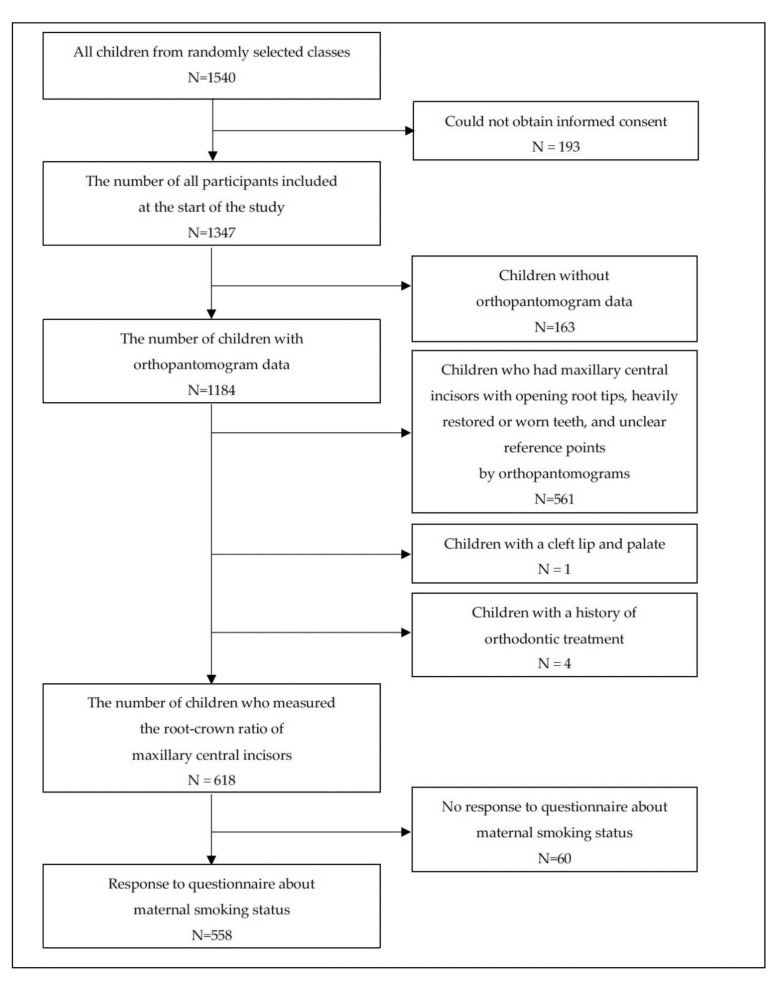
Flow chart of the sample selection process.

Of the 1540 selected children, 193 were excluded as informed consent could not be obtained from them (response rate: 87.5%). Of the remaining 1347 children, 163 who did not have orthopantomogram data were excluded. Furthermore, 561 children who had maxillary central incisors with opening root tips, heavily restored or worn teeth, and unclear reference points by orthopantomograms were excluded. Four children with a history of orthodontic treatment and one child with a cleft lip and palate were excluded. Information on maternal smoking status was missing for 60 participants. Hence, 558 children were included in the analysis (Figure 1). The age range was 8–16 years, with a mean age of 12.0 years.

### 2.3. Measurement of Root-Crown Ratio

The orthodontist (YS) with 2-year experience in orthodontic training at the Department of Maxillofacial Orthognathics, TMDU measured the R/C ratio of maxillary central incisors using orthopantomograms. A method developed by Lind [2] was used to measure root length and crown height using Image J software (NIH Image, Bethesda, MD, USA) [29]. A midpoint was visually determined on orthopantomograms along a line bisecting the mesial and distal cemento-enamel junctions (CEJs). Each root was measured from the apex to the corresponding midpoint. Each crown height was measured from the CEJ midpoints to the middle of the incisal edge. Data were compiled, and each root length was divided by the respective crown height to calculate the R/C ratio for each tooth. Additionally, the R/C ratio was calculated as the average value of the left and right teeth. For the ones whose one side could be measured only, that value was used. SRA was diagnosed when the R/C ratio of the maxillary central incisors was ≤1.0.

### 2.4. Maternal Smoking Status

We used responses to a self-reported questionnaire about maternal smoking status before and during pregnancy (current or not). Responses were selected based on the following options: (1) currently smoking; (2) quit smoking before pregnancy; (3) quit smoking when pregnant; (4) quit smoking during pregnancy but currently smoking; and (5) never smoked. We classified these variables into the following: ‘never smokers or former smokers not smoking since pregnancy (those who selected 2, 3, or 5)’, ‘current smokers not smoking during pregnancy (those who selected 4)’, and ‘current smokers who smoked during pregnancy (those who selected 1)’. Participants with missing information about maternal smoking status during pregnancy were excluded from the analyses.

### 2.5. Covariates

The following possible covariates were obtained from the questionnaire: sex of the child, paternal smoking during maternal pregnancy, gestational age (full term, <37 weeks, >42 weeks), family income level, maternal educational level, and maternal age at delivery (<20, 21–30, 31–40, >40 years).

Paternal smoking status was assessed using a questionnaire. The response was selected from the following options: (1) current smokers who smoked next to their spouse during pregnancy; (2) current smokers who did not smoke next to their spouse during pregnancy; (3) current smokers who did not smoke next to their spouse during pregnancy but smoked next to their family after birth; (4) current smokers who never smoked around their families; and (5) never smoked. We categorized these variables into ‘never smokers or current smokers not smoking around their family (those who selected 2, 4, or 5)’, ‘smokers who did not smoke next to their spouse during pregnancy but currently smoking around their family (those who selected 3)’, and ‘current smokers who smoked next to their spouse during pregnancy (those who selected 1)’.

Maternal educational level was assessed by the questionnaire and categorized into low level of education (‘no education was obtained’ and ‘obtained compulsory primary and/or lower secondary education’), intermediate level of education (‘completed high school’ and ‘completed vocational school’), and high level of education (‘completed a bachelor’s, master’s, or doctorate program). Family income level (measured in Mongolian Tughrik (MNT) and US dollar (USD)) was assessed using the questionnaire and categorized into low (<MNT 500,000), average (MNT 510,000−1,000,000), and high (>MNT 1,001,000 (USD 1 ~MNT 1437)).

### 2.6. Statistical Analysis

The associations between SRA and possible risk factors were analysed using multiple logistic regression. The regression model was adjusted for covariates, including the child’s sex, paternal smoking status, gestational age, family income level, maternal educational status, and maternal age at delivery. The significance level was set at *p* < 0.05. All analyses were performed using the Stata/SE 15.0 software package (Stata Corporation, College Station, TX, USA).

## 3. Results

A total of 558 children were included in this study. The mean R/C ratio of the maxillary central incisors was 1.21. It was larger in men than in women, but this difference was not statistically significant. The prevalence of SRA in these children was 14.2% (Table 1). Table 2 and Table 3 describe the demographic characteristics of the participants: sex, drinking habits of the mother during pregnancy, assessment of X-ray or vitamin supplements of the mother during pregnancy, birth weight, delivery, gestational age, family income level, educational level of mother or father, oral habits (finger sucking, open mouth, nail biting, lip sucking, object biting, bruxism), and history of facial trauma of their offspring. These variables were not significantly associated with SRA. 

The odds ratios (ORs) and 95% confidence intervals (CIs) for maternal factors associated with SRA are presented in Table 4. According to the crude model, children whose mothers were current smokers and smoked during pregnancy were more likely to show SRA (OR: 4.68, 95% CI: 1.72–12.72) than mothers who never smoked or were former smokers not smoking since pregnancy. After adjusting for possible confounders, children whose mothers were current smokers and smoked during pregnancy were found to be 4.95 times (95% CI: 1.65–14.79) more likely to present with SRA than those whose mothers had never smoked or were former smokers not smoking since pregnancy. In addition, fathers who were current smokers and smoked next to their spouse during pregnancy were found to be 1.86 times (95% CI: 1.02–3.40) more likely to have children with SRA than those fathers who never smoked or were current smokers but did not smoke around their family.

## 4. Discussion

This study examined the association between maternal or passive smoking during pregnancy and the development of SRA in maxillary central incisors. This study suggests that (1) maternal or passive smoking during pregnancy is significantly associated with SRA in maxillary central incisors, and (2) although root formation occurs after birth, it may be influenced by environmental factors in utero during pregnancy.

Previous studies have suggested that maternal smoking exposure during pregnancy is associated with missing teeth [19,20]. However, to the best of our knowledge, no population-based study has reported the association between maternal smoking exposure during pregnancy and tooth root formation. Therefore, the present study provides novel evidence to support that maternal smoking exposure during pregnancy is a risk factor for SRA in offspring. Children with mothers who were smoking during pregnancy were found to be more likely to show SRA. Tooth root formation of the central incisor is a developmental process that begins after birth. Thus, maternal smoking during pregnancy and/or passive smoking in children after birth can influence tooth root formation. In this study, current maternal smokers who did not smoke during pregnancy were not significantly associated with the development of SRA. Furthermore, fathers who were current smokers and smoked next to their spouse during pregnancy were more likely to have children with SRA. However, paternal smoking around the family after birth was not associated with SRA. Therefore, maternal smoking exposure during pregnancy is relevant to SRA in offspring.

Maternal and passive smoking during pregnancy can affect the formation of various organs in the foetus, such as the lungs, heart, kidneys, and palate, and tooth development [22]. Factors of smoking during pregnancy that may affect foetal organogenesis include nicotine [21] and epigenetic factors [30]. These factors may also affect root formation after birth. For example, epithelial-mesenchymal interactions are essential processes in organogenesis. Wnt/β-catenin signalling is an important pathway in this process and plays an important role in the lung development [31]. Disruption of this signalling causes long-term effects on postnatal lung development and asthmatic airway remodelling after birth [32]. Exposure to nicotine in the uterus due to smoking during pregnancy has been suggested to affect the activity of Wnt/β-catenin signalling [33]. Wnt/β-catenin signalling also regulates root formation [1]. Therefore, the effects of prenatal smoking exposure on tooth root formation may influence it after birth as well.

Epigenetic factors associated with smoking may also be involved in postnatal root formation. A recent study has suggested an association between maternal smoking during pregnancy and DNA methylation of EvC ciliary complex subunit 2 (*EVC2*) [30]. Mutations in *EVC2* cause congenital heart disease, shortened limbs, short stature, and dental abnormalities known as Ellis-van Creveld syndrome [34]. Previous studies have suggested that *EVC2* mutant mice have enamel hypoplasia and decreased root size in mandibular incisors [35]. The Evc/Evc2 protein complex is important for transducing Hedgehog signalling, which is involved in mediating epithelial-mesenchymal interactions to regulate root formation [35,36]. Therefore, DNA methylation of *EVC2* due to maternal smoking during pregnancy may also affect tooth root formation.

However, further studies are required to elucidate this mechanism in detail. In the future, we would like to conduct in vitro and in vivo experiments to study the mechanisms by which nicotine and epigenetic factors affect root formation.

The socio-economic environment of Mongolia has changed significantly since 1990 and has transitioned from a socialist to a democratic state [37]. In 1993, the first tobacco control law was enacted, but as of 2010, the smoking rate among males was approximately 46.3%, which is one of the highest worldwide. In contrast, women accounted for 6.8%, which was lower than that of the men [38]. However, since the Mongolian men are often at home, women and children are frequently exposed to passive smoking [39]. As shown in our study, passive smoking from fathers to pregnant mothers might increase SRA among children.

This study has several limitations. First, each questionnaire item was a self-reported response. Hence, self-reported exposure data may be subject to recall bias, which may have affected its validity. The questionnaire did not directly assess smoking during pregnancy. However, we defined those who selected ‘currently smoking’ as smokers during pregnancy in the present study, considering other response options. The responses were provided by recall sometime after the pregnancy, and details regarding the number of cigarettes smoked and specific duration of smoking were not collected. Second, other health behaviours, such as diet during pregnancy, were not evaluated. Thus, there may be residual confounding factors. Third, the number of mothers classified as current smokers who smoked during pregnancy was small. This may have resulted in wider CIs, making the results less precise. Fourth, only two public schools in the capital city were included in the study. Therefore, there is a possibility of sampling bias because the living environment of mothers may be different from that of the representative population. Finally, the diagnosis of SRA in this study was established only on maxillary central incisors, and the measurements were made using orthopantomograms, not from a three-dimensional perspective, which may have caused measurement errors.

## 5. Conclusions

Our findings suggest that maternal and passive smoke exposure during pregnancy can affect tooth root formation in children. Maternal smoking during pregnancy is particularly relevant to SRA in children. Further studies regarding the mechanism underlying the long-term effects of prenatal smoking exposure on tooth root formation in offspring are needed.

## Figures and Tables

**Table 1:** Comparison of the mean root-crown ratios of maxillary central incisors according to sex.

	ALL		Male		Female		*p* Value for *t*-Test
	(N = 558, 100%)		(N = 237, 42.5%)		(N = 321, 57.5%)		
	Mean	S.D.	Mean	S.D.	Mean	S.D.	
Root-crown ratio	1.21	0.18	1.23	0.19	1.20	0.18	0.09

Abbreviations: standard deviation, S.D.

**Table 2:** Socio-demographic characteristics of participants based on the presence of SRA.

Factors	All		SRA (−)		SRA (+)		*p* Value for the Chi-Squared Test	
	N = 558		N = 479 (85.8%)		N = 79 (14.2%)			
	N	%	N	%	N	%		
Sex of participants								
Male	237	42.5	207	43.2	30	38.0	0.38	
Female	321	57.5	272	56.8	49	62.0		
Drinking habit of mother during pregnancy								
(−)	502	96.5	429	96.4	73	97.3	0.68	
(+)	18	3.5	16	3.6	2	2.7		
X-ray of mother during pregnancy								
(−)	361	76.2	310	75.6	51	79.7	0.48	
(+)	113	23.8	100	24.4	13	20.3		
Mother taking vitamin supplements during pregnancy								
(−)	354	63.4	300	62.6	54	68.4	0.33	
(+)	204	36.6	179	37.4	25	31.6		
Birth weight								
Low (<2500 g)	16	3.3	13	3.1	3	4.3	0.60	
Normal (≥2500 g)	475	96.7	408	96.9	67	95.7		
Delivery								
Normal	428	80.0	374	81.1	54	73.0	0.20	
Difficult	26	4.9	20	4.4	6	8.1		
Caesarean	81	15.1	67	14.5	14	18.9		
Gestational age								
Pre-term (<37 weeks)	28	5.2	25	5.5	3	4.0	0.86	
Full term	444	83.2	380	83.0	64	84.2		
Post-term (>42 weeks)	62	11.6	53	11.5	9	11.8		
Family income level								
Low	165	26.6	139	30.0	26	35.1	0.36	
Average	281	55.3	248	53.4	33	44.6		
High	92	18.1	77	16.6	15	20.3		
Educational level of mother								
Low	109	20.0	98	20.9	11	14.9	0.48	
Intermediate	224	41.2	191	40.6	33	44.6		
High	211	38.8	181	38.5	30	40.5		
Educational level of father								
Low	130	25.8	111	25.6	19	26.4	0.98	
Intermediate	245	48.5	210	48.5	35	48.6		
High	130	25.7	112	25.9	18	25.0		

Abbreviations: short root anomaly, SRA.

**Table 3:** Characteristics of participants based on the presence of SRA.

Factors	All		SRA (−)		SRA (+)		*p* Value for the Chi-Squared Test	
	N = 558		N = 479 (85.8%)		N = 79 (14.2%)			
	N	%	N	%	N	%		
Finger sucking habit								
(−)	504	92.8	435	92.9	69	92.0	0.77	
(+)	39	7.2	33	7.1	6	8.0		
Open mouth habit								
(−)	502	90.1	432	90.4	70	88.6	0.63	
(+)	55	9.9	46	9.6	9	11.4		
Nail biting habit								
Never	445	82.1	386	82.7	59	78.7	0.13	
Did before	55	10.2	49	10.5	6	8.0		
Still does	42	7.7	32	6.8	10	13.3		
Lip sucking habit								
Never	507	92.8	439	93.6	68	88.3	0.12	
Did before	25	4.6	18	3.8	7	9.1		
Still does	14	2.6	12	2.6	2	2.6		
Object biting habit								
Never	427	78.8	369	79.0	58	77.3	0.84	
Did before	80	14.8	69	14.8	11	14.7		
Still does	35	6.4	29	6.2	6	8.0		
Bruxism								
Never	456	84.8	390	84.2	66	88.0	0.63	
Did before	43	8.0	39	8.4	4	5.3		
Still does	39	7.2	34	7.4	5	6.7		
History of facial trauma								
(−)	484	88.6	413	87.7	71	94.7	0.08	
(+)	62	11.4	58	12.3	4	5.3		

**Table 4:** Unadjusted and adjusted odds ratios for SRA.

Factors	SRA (−)	SRA (+)	Crude		Adjusted	
	N = 479 (85.8%)	N = 79 (14.2%)	OR (95 % CI)	*p* Value	OR (95 % CI)	*p* Value
Maternal smoking status						
Never or former smokers who quit smoking before/at pregnancy	448	67	Ref		Ref	
Current smokers who did not smoke during pregnancy	21	5	1.59 (0.58–4.36)	0.37	1.48 (0.52–4.21)	0.47
Current smokers who smoked during pregnancy	10	7	4.68 (1.72–12.72)	0.002	4.95 (1.65–14.79)	0.004
Sex of participants						
Male	207	30	Ref		Ref	
Female	272	49	1.24 (0.76–2.03)	0.38	1.20 (0.72–2.02)	0.49
Paternal smoking status						
Never or current smokers not smoking around their family	368	52	Ref		Ref	
Current smokers who did not smoke next to their spouse during pregnancy but were smoking around their family after birth	13	2	1.09 (0.24–4.96)	0.91	1.39 (0.29–6.61)	0.68
Current smokers who smoked next to their spouse during pregnancy	74	22	2.10 (1.20–3.67)	0.009	1.86 (1.02–3.40)	0.04
Missing	24	3	0.88 (0.26-3.04)	0.85	0.81 (0.22-3.01)	0.76
Gestational age						
Full term	380	64	Ref		Ref	
Pre-term (<37 weeks)	25	3	0.71 (0.21–2.43)	0.59	0.71 (0.20–2.51)	0.60
Post-term (>42 weeks)	53	9	1.01 (0.47–2.14)	0.98	1.02 (0.46–2.24)	0.97
Missing	21	3	0.85 (0.25–2.93)	0.79	0.58 (0.15–2.22)	0.42
Family income level						
Low	139	26	Ref		Ref	
Average	248	33	0.71 (0.41–1.24)	0.23	0.68 (0.37–1.24)	0.21
High	77	15	1.04 (0.52–2.08)	0.91	1.00 (0.46–2.20)	0.99
Missing	15	5	1.78 (0.60–5.33)	0.30	1.47 (0.37–5.87)	0.59
Educational level of mother						
Low	98	11	Ref		Ref	
Intermediate	191	33	1.54 (0.75–3.18)	0.24	1.77 (0.83–3.78)	0.14
High	181	30	1.48 (0.71–3.07)	0.30	1.86 (0.82–4.21)	0.14
Missing	9	5	4.95 (1.41–17.42)	0.01	4.52 (0.92–22.26)	0.06
Maternal age at delivery						
≤19 years	47	7	Ref		Ref	
20–29 years	325	56	1.16 (0.50–2.69)	0.74	1.22 (0.50–2.99)	0.66
≥30 years	94	14	1.00 (0.38–2.64)	1.00	0.90 (0.32–2.51)	0.85
Missing	13	2	1.03 (0.19–5.58)	0.97	0.72 (0.10–5.30)	0.75

Abbreviations: odds ratio, OR. confidence interval, CI.

## Data Availability

The datasets used in this study are available from the corresponding author on reasonable request.
